# Post COVID-19 condition and health-related quality of life: a longitudinal cohort study in the Belgian adult population

**DOI:** 10.1186/s12889-023-16336-w

**Published:** 2023-07-27

**Authors:** Pierre Smith, Robby De Pauw, Dieter Van Cauteren, Stefaan Demarest, Sabine Drieskens, Laura Cornelissen, Brecht Devleesschauwer, Karin De Ridder, Rana Charafeddine

**Affiliations:** 1 Department of Epidemiology and Public Health, Sciensano, Rue Juliette Wytsmanstraat 14, Brussels, 1050 Belgium; 2 Institute of Health and Society (IRSS), Université catholique de Louvain, Brussels, Belgium; 3 Department of Rehabilitation Sciences, Ghent University, Ghent, Belgium; 4 Department of Translational Physiology, Infectiology and Public Health, Ghent University, Ghent, Belgium

**Keywords:** COVID-19, Post COVID-19 condition, Health-related quality of life, HRQoL, Longitudinal study

## Abstract

**Background:**

Since the onset of the COVID-19 pandemic, most research has focused on the acute phase of COVID-19, yet some people experience symptoms beyond, referred to as post COVID-19 conditions (PCC). However, evidence on PCC and its impacts on health-related quality of life (HRQoL) is still scarce. This study aimed to assess the impact of COVID-19 and PCC on HRQoL.

**Methods:**

This is a longitudinal cohort study of the Belgian adult population with recent SARS-CoV-2 infection. In total, 5,727 people were followed up between the time of their infection and three months later. HRQoL was measured with the EQ-5D-5L questionnaire before and during the infection and three months later. Linear mixed regression models were built to assess the longitudinal association between participants’ characteristics and the evolution of their HRQoL.

**Results:**

This study found a significant decline in HRQoL during the SARS-CoV-2 infection in comparison to the situation before (β=-9.91, 95%CI=-10.13;-9.85), but no clinically important difference three months after the infection compared to the situation before, except among people reporting PCC (β=-11.15, 95%CI=-11.72;-10.51). The main symptoms of PCC with a significant negative impact on the different dimensions of HRQoL were fatigue/exhaustion (21%), headache (11%), memory problems (10%), shortness of breath (9%), and joint (7%) or muscle pain (6%). The dimension of HRQoL most negatively affected by several PCC symptoms was pain/discomfort.

**Conclusions:**

With the growing number of people infected with SARS-CoV-2, PCC and its impact on HRQoL are becoming important public health issues. To allow people with PCC to recover and to limit its detrimental impact on HRQoL, it is essential to manage its various heterogeneous symptoms using a multidisciplinary approach.

**Supplementary Information:**

The online version contains supplementary material available at 10.1186/s12889-023-16336-w.

## Background

In December 2019, the SARS-CoV-2 virus originated in China, and soon the COVID-19 pandemic affected lives on a worldwide scale. Belgium has been harshly affected by the virus in terms of cases, hospitalisations and deaths [1]. During the first peak of the pandemic in March and April 2020, Belgium faced high COVID-related mortality and the situation in nursing homes was critical [2, 3]. A consequence of the rapid spread of the virus and its clinical manifestations is that most of the research has focused on managing the acute symptoms of the disease [4, 5]. However, there is growing evidence that SARS-CoV-2 infection may have longer-term effects even after recovery from the acute infection [6]. This phenomenon was defined by the World Health Organization (WHO) as post COVID-19 condition (PCC) or long COVID [7].

Initially, attention to PCC was drawn by patients who called themselves “long haulers”. They were still suffering from symptoms months after recovering from the acute phase of their SARS-CoV-2 infection, with a significant impact on their daily lives [6]. In October 2021, the WHO published the following clinical case definition of PCC using Delphi methodology with patients and experts: *“Post COVID-19 condition occurs in individuals with a history of probable or confirmed SARS-CoV-2 infection, usually 3 months from the onset of COVID-19 with symptoms and that last for at least 2 months and cannot be explained by an alternative diagnosis.”* [7]. The types of persistent symptoms, their prevalence, duration, and mechanisms are not well understood and still under investigation. In terms of PCC prevalence, a recent meta-analysis [8] carried out on 33 studies on hospitalised and non-hospitalised COVID-19 survivors showed that 46% of the them had at least one symptom three months after acute infection. Another meta-analysis found that the most common symptoms of PCC were fatigue (58%), headache (44%), attention disorder (27%) and dyspnoea (24%) [9]. Finally, some studies have also shown that PCC is not evenly distributed among people infected with SARS-CoV-2, with a higher risk among women [10], people with pre-existing comorbidities [11], and people hospitalised due to COVID-19 [12]. These data show on the one hand that PCC is becoming an important public health issue, and on the other hand that the symptoms of PCC can lead to disabilities in daily life with an impact on the quality of life of those who suffer from it.

Health-related quality of life (HRQoL) is a comprehensive and relevant indicator for assessing the impact of a disease or disability on the physical, mental and social domains of individual health. A meta-analysis on 12 studies (follow-up time from SARS-CoV-2 infection between 30 and 180 days) assessing the impact of PCC on HRQoL showed that 59% of people with PCC reported a worsening in quality of life [13]. This meta-analysis also concluded that further research was needed as most of the included studies involved only people hospitalised due to COVID-19 and were not based on population-wide sampling. However, PCC also affects people with moderate acute symptoms not requiring hospitalisation and even people who were asymptomatic during the acute phase of their SARS-CoV-2 infection [11, 14, 15].

This longitudinal cohort study in the Belgian adult population aimed [1] to assess the impact of a SARS-CoV-2 infection and PCC on HRQoL at the time of infection and after three months, and [2] to identify the sociodemographic and clinical factors associated with the evolution of HRQoL following the infection.

## Methods

### Setting

Since the start of the pandemic until February 2022, Belgium faced five waves of the COVID-19 pandemic [16]. Data for this study were collected between April 29, 2021 and May 1, 2022. This period corresponds to the end of the third wave (Alpha variant, March-April 2021), the fourth peak (Delta variant, October-December 2021), and the fifth peak of the pandemic (Omicron BA.1 variant, January-February 2022). The roll-out of COVID-19 vaccination was initiated in January 2021. When the study was launched in April 2021, the vaccination rate in the adult population was around 6% and by May 2022 it reached 78% for a complete primary vaccination schedule (1 or 2 doses, according to brand) [17].

### Study design and population

The detailed study protocol (i.e. flowchart, response rate, loss to follow-up, etc.) has been published elsewhere [18]. In short, this prospective longitudinal cohort study includes two online questionnaires: a baseline questionnaire sent to participants at the time of their confirmed SARS-CoV-2 infection and a follow-up questionnaire sent three months later. The baseline questionnaires were sent between April 29, 2021 and February 1, 2022, and the follow-up questionnaires between July 29, 2021 and May 1, 2022. In total, 5,727 people completed both questionnaires between April 29, 2021 and May 1, 2022.

The eligible population were people aged 18 years and older, living in Belgium, with a recent SARS-CoV-2 infection confirmed via a molecular or antigen test (*n* = 1,412,208, from April 29, 2021 to May 1, 2022). In Belgium, the test results from molecular and antigen tests are send to a central database at Sciensano (the Belgian Institute for Health) [19, 20]. Contact tracing call centers used these test results to contact COVID-19 cases and trace their contacts. At the end of the call, the contact tracing agents informed the eligible cases about the COVIMPACT study and asked them if they agreed to receive a link to the first online questionnaire. Follow-up questionnaires were emailed to participants three months after their inclusion in the study. The published study protocol [18] showed that the follow-up participation rate was 79%, and that the proportion of people between 46-65 years, of women, and of people reporting at least one acute COVID-19 symptom was higher among cohort participants than in the eligible population, resulting in sample selection bias. Therefore, post-stratification weights were used to adjust for the distribution of the eligible population (see statistical analysis).

### Measures

The English version of the questions asked to assess the main outcomes of this study are presented in the supplementary materials (Additional file 1). The primary outcome of this study was the HRQoL of participants. This was assessed using the self-administrated EuroQol 5-dimensional-5 levels (EQ-5D-5 L) questionnaire developed by the EuroQol Group in 2011 and subsequently validated and widely used [21, 22]. It covers five domains (mobility, self-care, usual activities, pain or discomfort, and anxiety or depression). Each domain has five levels (no problems, some problems, moderate problems, severe problems, and extreme problems/unable to). For each possible EQ-5D-5L health state, an index value can be calculated based on country-specific value sets. In this study, we used the most recent (2018) value set for Belgium [23] ranging between --0.53 (worst health state) and 1 (most optimal health state). The HRQoL of participants was measured for three periods with the baseline questionnaire to assess the situation [1] before the infection (retrospectively), [2] during the acute phase of the infection, and [3] three months after infection with the follow-up questionnaire. In several studies, the EQ-5D-5 L is accompanied by the EQ-VAS, a vertical visual analogue scale to assess self-rated health. This scale was not included in the questionnaires in order to limit the number of questions and the time to complete them.

The following independent variables were included in the analyses, in line with the existing literature on PCC and COVID-19 [11, 12, 24, 25]: age, sex, educational status, having a chronic disease, body mass index, COVID-19 vaccination status at the time of infection, number of symptoms in the acute phase of the infection, whether or not they have been hospitalised following the SARS-CoV-2 infection, and whether or not they have PCC.

PCC was defined on the basis of the guidelines of the National Institute for Health and Care Excellence (NICE) [26] as having at least one symptom related to SARS-CoV-2 infection three months after it. Several studies have shown that people with PCC tend to report many and heterogeneous symptoms [8, 27]. Therefore, it is important to have the most comprehensive list of symptoms to assess the presence of PCC. In this study, a list of 30 potential symptoms of PCC (See Table 4) was used based on published guidelines [7, 26, 28, 29]. The question asked in the three-month follow-up questionnaire was: *“Within the last seven days have you had any of these symptoms? (that you did not experience before onset of your COVID-19 illness)”.*

All these variables were self-reported.

### Statistical analysis

Post-stratification weights were used to adjust for the distribution of the eligible population. The variables available for the eligible population (data from national tracing centers) that were included for the calculation of post-stratification weights were age, sex, and having at least one acute symptom of COVID-19.

Descriptive statistics were computed for socio-demographic and clinical characteristics of the cohort participants. Regarding statistical analysis on HRQoL scores, a previous study showed that the use of linear regressions was appropriate [30]. The EQ-5D-5L scores (HRQoL scores) were reported as mean (SD) for the whole sample and for the three time periods: before infection, at the time of infection, and three months later. The minimal detectable change (MDC) of the HRQoL score before infection was assessed using a commonly used distribution-based approach: the standard error of the measure (SEM) [31]. The MDC can be interpreted as the magnitude of change below which there is more than a 95% chance that no real change in the HRQoL score has occurred. At the 95% confidence level, MDC = SEM * √2 * 1.96. The SEM is calculated as ${\sigma }_{x}*\sqrt{1-{r}_{x}}$ where r is the reliability of the measure. One study on people with PCC found that the test-retest reliability of the EQ-5D-5L was 0.86 [32], so we used that value.

Additional analyses were performed to estimate the proportion of people three months after infection with problems in the five dimensions of HRQoL (no problem vs. some to severe problems) and explore the effect of PCC on HRQoL by comparing people with and without protracted symptoms. Data of the 2018 Belgian Health Interview Survey (BHIS) [33] were used to compare HRQoL in the Belgian adult population without COVID-19 (*n* = 12,742). A sub-sample (*n* = 3,263) was extracted from the BHIS using a stratified random sampling method to match the distribution of age, sex and level of education of the present study cohort, to have a matched non-COVID-19 control group.

HRQoL being evaluated three times for each participant (before infection, during infection, after three months, measurements within the same person are correlated, violating the assumption of conditionally independent observations in regression models. Therefore, the current study uses linear mixed regression models to assess changes in HRQoL scores, which has been shown to be an appropriate statistical methodology [30]. The models performed allow to assess the strength of the association between the different sociodemographic and clinical characteristics of participants and the evolution of their HRQoL over time. The linear mixed models used provide estimates for the different covariates (i.e. fixed component of the model) taking into account the clustering effect due to each participant (i.e. random component). We used ANOVA tests to estimate the overall effect of categorical variables. The modelling process was in three steps. First, univariate linear mixed models were performed to test the association between each potential predictor and HRQoL. Second, a multivariable linear mixed model was performed with only the significant factors in the univariate regressions (*p* < 0.05). Third, the association between PCC and HRQoL may differ depending on the following participants’ factors that may influence both: the vaccination status, the number of symptoms during the acute phase of infection, and whether or not they have been hospitalised following the SARS-CoV-2 infection. Interactions were, therefore, computed and included one by one in the model to assess whether these factors had a moderating effect on the association between PCC and HRQoL. Only the significant interaction effects were included in the final model. The Akaike Information Criterion (AIC) was used to select the model and co-variance structure with the best fit (lowest value). In the final model, we used the Compound Symmetric (CS) structure for the variance covariance matrix.

Finally, ordinal logistic regression models were also performed to assess the association between the different symptoms of PCC and the five ordinal dimensions of HRQoL three months after the SARS-CoV-2 infection (five ordinal categories in each dimensin, from no problems to extreme problems/unable to). The results are presented as odds ratio with 95% confidence intervals.

All statistical analysis were performed in SAS® 9.4.

## Results

Table 1 presents the sociodemographic characteristics of the cohort.

**Table 1:** Sociodemographic and clinical characteristics of the cohort

	n (%) *weighted %*
Whole sample	*n* = 5,727
Age	
• 18–25	573 (10.0) *13.8*
• 26–45	2726 (47.6) *53.5*
• 46–65	2164 (37.8) *26.9*
• 66 +	264 (4.6) *5.8*
Sex	
• Men	2027 (35.4) *43.9*
• Women	3700 (64.6) *56.1*
Educational level	
• Secondary school or below	1628 (28.4) *29.6*
• Higher education	4099 (71.6) *70.4*
Chronic disease	
• Yes	412 (7.2) *7.4*
• No	5315 (92.8) *92.6*
Body Mass Index (BMI)	
• Normal (18.5–24.9)	2486 (43.4) *44.9*
• Over-weight (25.0–29.9)	2039 (35.6) *33.3*
• Obesity (≥ 30.0)	1202 (21.0) *21.8*
COVID-19 vaccination status at the time of infection	
• None	1850 (32.3) *41.6*
• Partial	681 (11.9) *11.8*
• Complete primary schedule	3196 (55.8) *46.6*
Acute COVID-19 symptoms	
• None	349 (6.1) *10.1*
• 1–4	1660 (29.0) *31.4*
• 5–8	2068 (36.1) *32.1*
• > 8	1650 (28.8) *26.4*
Hospitalisation following COVID-19	
• Yes	109 (1.9) *2.5*
• No	5618 (98.1) *97.5*
Post COVID-19 Condition	
• Yes	2829 (49.4) *46.7*
• No	2898 (50.6) *53.2*

The cohort was largely composed of people aged 26 to 45 (48%), women (65%), and people with a higher level of education (72%). In terms of health, 7% had a chronic disease and 21% suffered from obesity (BMI ≥ 30). Regarding COVID-19 variables, 56% of the people had completed primary vaccination at the time of their infection, 36% had between four and eight acute COVID-19 symptoms, and 2% were hospitalised. Finally, 49% of the cohort had PCC (i.e. reported at least one symptom related to their SARS-CoV-2 infection three months afterwards).

Table 2 presents the evolution of HRQoL scores. The weighted mean HRQoL score before the SARS-CoV-2 infection was 0.92 (95%CI = 0.917;0.923), it dropped to 0.80 (95%CI = 0.795;0.805) at the time of infection, and increased to 0.91 (95%CI = 0.905;0.911) after three months. For comparison, the mean HRQoL score in the matched non-COVID-19 control group (BHIS 2018) sample was 0.91 (95%CI = 0.906;0.914). The minimal detectable change (MDC) criterion was estimated based on the HRQoL score before SARS-CoV-2 infection for the whole sample. The decrease in HRQoL score at time of infection was greater than the MDC, which suggests that the decrease was clinically observable. In contrast, the decrease in HRQoL three months after infection, compared to before infection, is smaller than the MDC and hence probably not clinically relevant.

**Table 2:** Evolution of Health-Related Quality of Life (HRQoL) scores

Whole sample *n* = 5,727	HRQoL before infection (a)	HRQoL at time of infection (b)	HRQoL 3 months after infection (c)
	Weighted Mean (SD)	Weighted Mean (SD)	Weighted Mean (SD)
	0.92 (0.11)	0.80 (0.18)	0.91 (0.13)
Minimal detectable change (MDC)^a^	0.113	Meets MDC criterion	Does not meet MDC criterion

^a^At the 95% confidence level, MDC = SEM × √2 × 1.96, with the SEM = ${\sigma }_{x}*\sqrt{1-{r}_{x}}$, (r_x_ = 0.86)

**Fig. 1:**
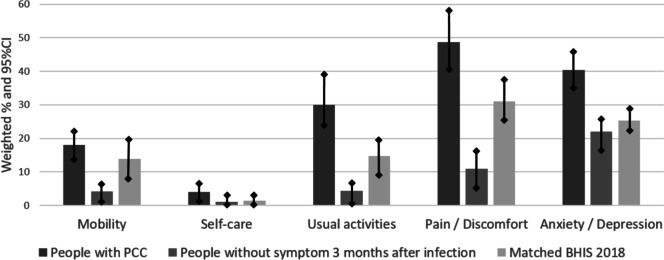
Weighted proportion of people reporting problems in the five dimensions of health-related qualiy of life (HRQoL) three months after SARS-CoV-2 infection, comparing people with post COVID-19 conditions (PCC) with people without symptom after three months, and with a matched non-COVID-19 control group (BHIS 2018)

Figure 1 presents the proportion of people reporting some or severe problems in the five dimensions of HRQoL three months after SARS-CoV-2 infection, comparing people with PCC with people without symptoms after three months, and with the matched non-COVID-19 control group (BHIS 2018).

The proportion of people reporting problems was significantly different between people with PCC and those without symptoms after three months in each of the five dimensions of HRQoL. In all dimensions, the proportion was the highest in people with PCC, followed by the BHIS 2018 control group, and people without symptoms.

The pain/discomfort dimension had the highest proportion of people reporting problems three months after infection, with 49% in people with PCC, 11% in people without symptom three months after their SARS-CoV-2 infection, and 31% in the BHIS 2018 control group. The second dimension with the highest proportion was anxiety/depression, with 40% in people with PCC and 22% in people without symptom, and 25% in the BHIS 2018 control group. The dimension with the lowest proportion was self-care, with 4% in people with PCC and 1% in people without symptoms (BHIS 2018 = 1.4%).

Table 3 presents the association between the change in HRQoL following COVID-19 (i.e. before infection, during infection, and after three months) and the sociodemographic and clinical characteristics of the cohort. The AIC value was lower in the final model with the interaction term, indicating better goodness of fit. The multivariable linear mixed model showed a statistically significant effect of time on the change in HRQoL score, with a lower score during the SARS-CoV-2 infection (β=-9.91; *p* < 0.001) and three months after (β=-2.61; *p* < 0.001), in comparison to the score before infection. Regarding the sociodemographic factors, women (β=-2.17; *p* < 0.001) and people with a lower educational level (β=-1.43; *p* < 0.001) were more likely to have a decrease over time in HRQoL. A decrease over time in HRQoL was also found in people with a chronic disease (β=-7.22; *p* < 0.001), and in people with obesity compared to people with a normal BMI (β=-1.58; *p* < 0.001). Regarding factors related to the acute phase of COVID-19, people without COVID-19 vaccination at the time of infection were more likely to have a decrease in HRQoL score compared to people with a complete primary schedule (β=-1.19; *p* = 0.006) as well as people hospitalised following their infection (β=-1.68; *p* = 0.01). The number of acute COVID-19 symptoms at the time of infection was also associated with a decrease in HRQoL score (β=-7.21; *p* < 0.001).

**Table 3:** Association between change in health-related quality of life (HRQoL) following COVID-19 and sociodemographic characteristics of the cohort (linear mixed models with HRQoL before infection, during infection, and after three months)

	**Change in HRQoL**^a^		
	Univariate regression models	Multivariable regression model	Multivariable regression model with interaction term(s)^b^
	β (95% CI) *p*-value	β (95% CI) *p*-value	β (95% CI) *p*-value
Time, (REF = before infection)			
• During infection	-9.97 (-10.11; -9.83) < 0.001	-9.90 (-10.05;- 9.74) < 0.001	-9.91 (-10.13;-9.85) < 0.001
• After three months	-0.67 (-0.97;-0.36) < 0.001	-2.57 (-3.62;-1.51) < 0.001	-2.61 (-3.66;-1.55) < 0.001
* Overall effect*	*p* < *0.001*		*p* = *0.003*
Age, (REF = 26–45)		/	/
• 18–25	-0.12 (-0.49;0.25) 0.52		
• 46–65	-0.16 (-0.56;0.22) 0.39		
• 66 +	0.14 (-0.11;0.40) 0.26		
* Overall effect*	*p* = *0.15*		
Sex, Women (REF = Men)	-3.71 (-3.92;-3.49) < 0.001	-2.16 (-2.36;-1.96) < 0.001	-2.17 (-2.32;-1.80) < 0.001
Educational level, Secondary school or below (REF = Higher)	-1.87 (-2.12;-1.61) < 0.001	-1.43 (-1.65;-1.20) < 0.001	-1.43 (-1.66;-1.20) < 0.001
Chronic disease, Yes (REF = No)	-5.52 (-6.22;-4.49) < 0.001	-7.39 (-10.99;-3.80) < 0.001	-7.22 (-7.82;-6.61) < 0.001
Body Mass Index, (REF = Normal, BMI = 18.5–24.9)			
• Overweight (BMI = 25.0–29.9)	-0.22 (-0.45;0.01) 0.05	-0.08 (-0.30;0.12) 0.41	-0.02 (-0.27;0.15) 0.61
• Obesity (BMI ≥ 30.0)	-2.62 (-2.94;-2.31) < 0.001	-1.58 (-1.87;-1.29) < 0.001	-1.58 (-1.87;-1.29) < 0.001
* Overall effect*	*p* = *0.08*	*p* = *0.08*	*p* = *0.09*
COVID-19 vaccination status at the time of COVID-19 infection, (REF = Complete primary schedule)			
• None	-1.98 (-2.35;- 1.62) < 0.001	0.005	-1.19 (-1.87;-0.42) 0.006
• Partial	-1.73 (-2.49;-0.98) < 0.001	-1.06 (-1.40;-0.73) 0.002	-0.88 (-1.81;0.05) 0.07
* Overall effect*	*p* < *0.001*	*p* = *0.006*	*p* = *0.02*
Number of acute COVID-19 symptoms	-5.61 (-6.89;-3.29) < 0.001	-6.38 (-6.95;-5.27) < 0.001	-7.21 (-7.93;-6.22) < 0.001
Hospitalisation following COVID-19, Yes (REF = No)	-7.37 (-10.97;-3.78) < 0.001	-3.31 (-6.71;-0.08) 0.04	-1.68 (-2.98;-0.38) 0.01
Post COVID-19 condition, Yes (REF = No)	-13.46 (-14.03;-12.59) < 0.001	-11.26 (-11.89;-10.64) < 0.001	-11.15 (-11.72;-10.51) < 0.001
PCC^a^ Hospitalisation			
• Yes^a^ Yes	/	/	-5.78 (-11.20;-0.35) 0.03
• Yes^a^ No (REF)			
• No (REF)^a^ Yes			
• No (REF)^a^ No			
* Model goodness of fit: AIC*	/	*5393.9*	*5165.2*

^a^The HRQoL scores were multiplied by 100 for the modeling to avoid too many decimals in the results

^b^Interactions between PCC and the vaccination status and number of symptoms during the acute phase of infection were not significant

Having PCC three months after SARS-CoV-2 infection was also associated with a decrease over time in HRQoL score (β=-11.15; p < 0.001). In addition, the final model showed a significant interaction between PCC and hospitalisation, showing that the negative association between PCC and HRQoL was stronger among participants hospitalised (β=-5.78; *p* = 0.03).

**Fig. 2:**
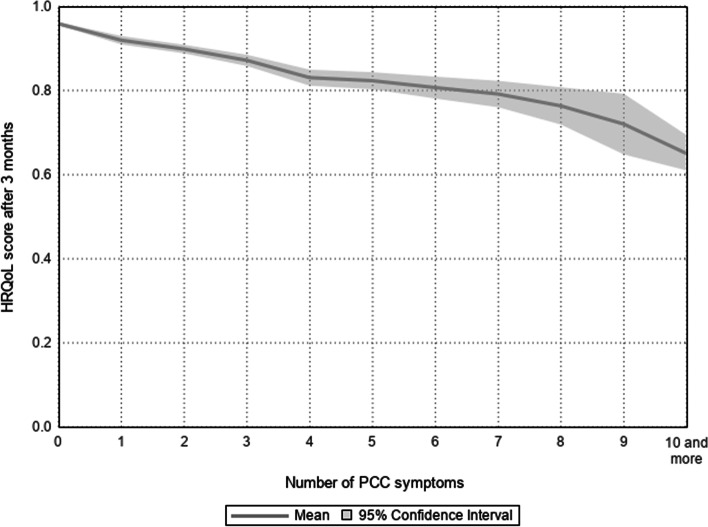
Health-related qualiy of life (HRQoL) score three months after SARS-CoV-2 infection according to the number of post COVID-19 conditions (PCC) symptoms reported, mean and 95% confidence interval

 Figure 2 presents the mean and 95% confidence interval of the HRQoL score three months after the SARS-CoV-2 infection according to the number of PCC symptoms reported. The figure shows a gradual decrease in the HRQoL score with increasing number of PCC symptoms, with people reporting no PCC symptoms having a mean score 0.95 (95%CI = 0.95–0.96) and those reporting 10 or more PCC symptoms a mean score of 0.65 (95%CI = 0.61–0.69).

Table 4 presents the associations between the self-reported symptoms of PCC and having a problem in each of the five dimensions of HRQoL three months after SARS-CoV-2 infection. The most frequent self-reported PCC symptoms in the cohort (weighted %) were fatigue/exhaustion (21%), headache (11%), memory problems (10%), loss of smell and/or taste (10%), shortness of breath (9%), and sleeping problems (8%).

**Table 4:** Self-reported symptoms of post COVID-19 condition associated with problems in each of the five dimensions of HRQoL three months after SARS-CoV-2 infection

**Symptoms of PCC 3 months after infection**	Weighted %	Multivariable ordinal regression models^a^ OR (95% CI)				
		Problems in mobility	Problems in self-care	Problems in usual activities	Problems in Pain / Discomfort	Problems in Anxiety / Depression
Fatigue/exhaustion	20.7	**1.53 (1.16;2.33)**	1.35 (0.72;1.97)	**3.12 (2.39;4.64)**	1.33 (0.86;1.15)	**2.25 (1.72;3.77)**
Headache	10.8	0.93 (0.78;1.24)	1.32 (0.87;2.77)	1.13 (0.68;2.58)	**2.15 (1.73;2.80)**	1.16 (0.71;1.81)
Memory problems	10.4	**1.28 (1.15;1.61)**	1.09 (0.46;1.22)	**3.39 (2.26;4.52)**	1.06 (0.73;1.19)	**1.98 (1.25;2.40)**
Loss of smell and/or taste	10.2	1.03 (0.71;1.39)	0.73 (0.27;1.49)	1.12 (0.46;1.78)	1.19 (0.73;1.95)	0.75 (0.29;1.81)
Muscle pain	6.5	**1.33 (1.17;1.42)**	**1.78 (1.23;2.93)**	**2.31 (1.16;3.33)**	**3.14 (2.19;4.11)**	1.31 (0.76;2.65)
Shortness of breath	8.7	**2.24 (1.15;2.63)**	**2.11 (1.72;2.61)**	**1.95 (1.66;3.24)**	**2.19 (1.91;2.88)**	**1.75 (1.46;2.74)**
Sleeping problems	8.3	1.09 (0.87;1.31)	1.03 (0.91;1.35)	**2.14 (2.02;2.86)**	1.05 (0.93;1.67)	**2.20 (1.69;2.91)**
Joint pain	6.7	1.41 (0.78;1.94)	0.96 (0.43;1.89)	1.16 (0.33;1.71)	**3.57 (3.04;4.40)**	1.12 (0.59;1.85)
Dizziness	4.9	1.12 (0.63;1.41)	1.16 (0.87;1.65)	**1.53 (1.24;2.92)**	**1.51 (1.22;2.32)**	1.09 (0.74;1.88)
Palpitations	4.8	1.02 (0.74;1.31)	0.85 (0.67;1.41)	1.02 (0.44;1.80)	1.16 (0.58;1.64)	0.98 (0.63;1.76)
Persistent cough	4.2	1.06 (0.96;1.88)	1.41 (0.79;2.93)	0.98 (0.46;1.73)	**1.54 (1.02;2.86)**	1.05 (0.43;1.87)
Constipation	3.8	1.16 (0.81;1.32)	1.24 (0.48;1.80)	1.03 (0.67;1.89)	1.09 (0.53;1.35)	0.87 (0.31;1.73)
Problems seeing	3.7	0.89 (0.58;1.41)	0.85 (0.48;1.66)	0.89 (0.48;1.70)	1.20 (0.79;2.41)	1.01 (0.60;2.32)
Chest pain	3.6	**1.25 (1.07;1.63)**	1.64 (0.76;2.92)	**2.55 (1.47;3.83)**	**2.55 (1.32;3.94)**	1.15 (0.47;2.73)
Ringing in ears	3.2	1.06 (0.81;1.28)	0.92 (0.67;1.07)	1.20 (0.96;1.45)	1.10 (0.95;1.65)	1.02 (0.57;1.19)
Tingling feeling	3.1	0.97 (0.64;1.28)	1.33 (0.61;2.29)	0.93 (0.37;2.69)	0.88 (0.42;1.84)	0.85 (0.16;1.91)
Loss of appetite	2.7	1.21 (0.73;1.63)	1.19 (0.48;1.80)	0.70 (0.29;1.31)	0.95 (0.54;2.36)	1.22 (0.81;2.63)
Stomach pain	2.6	0.69 (0.27;1.23)	1.41 (0.67;3.15)	0.82 (0.28;1.30)	**2.11 (1.37;3.12)**	1.15 (0.41;1.90)
Skin rashes	2.6	0.85 (0.79;1.22)	1.02 (0.66;2.38)	0.93 (0.57;2.29)	1.10 (0.74;1.86)	0.71 (0.35;1.17)
Others	2.4	1.09 (0.87;2.31)	1.38 (0.86;2.30)	1.06 (0.34;2.18)	1.06 (0.24;3.98)	0.55 (0.20;2.31)
General malaise	2.0	0.88 (0.45;3.11)	1.31 (0.85;2.77)	0.87 (0.41;2.33)	1.03 (0.57;1.99)	1.16 (0.79;2.62)
Weight loss	2.0	0.77 (0.44;2.12)	0.65 (0.09;1.21)	1.15 (0.59;1.71)	0.80 (0.24;1.36)	0.69 (0.13;1.25)
Confusion	1.7	1.13 (0.81;3.35)	0.91 (0.49;2.13)	2.31 (2.09;4.53)	0.98 (0.76;1.61)	1.10 (0.88;2.32)
Problems speaking	1.6	1.78 (0.77;3.69)	1.79 (0.88;3.73)	1.03 (0.12;2.94)	1.19 (0.28;2.66)	1.07 (0.16;2.98)
Problems swallowing	0.2	0.86 (0.47;2.42)	1.85 (0.83;3.17)	1.32 (0.60;2.44)	2.11 (0.99;4.23)	0.72 (0.21;2.14)
Swelling/oedema	0.1	1.29 (0.61;2.28)	1.72 (0.73;3.71)	0.95 (0.51;2.66)	1.85 (0.34;3.56)	1.03 (0.31;3.21)
Incontinence	0.1	1.12 (0.43;3.01)	1.35 (0.82;2.87)	0.71 (0.28;2.34)	1.14 (0.51;2.77)	0.98 (0.35;1.91)

^a^Adjusted for the different independent variables in Table 3

Having shortness of breath was associated with problems in each of the five dimensions of HRQoL three months after the SARS-CoV-2 infection. Fatigue/exhaustion and having memory problems were associated with problems in the dimensions of mobility, usual activities, and anxiety/depression. Having muscle pain was associated with problems in the dimensions of mobility, usual activities, and pain/discomfort. The following PCC symptoms were aslso associated with having problems in usual activities: sleeping problems, dizziness, and chest pain. Several other PCC symptoms were also significantly associated with problems in the dimension of pain/discomfort: headache, joint pain, dizziness, persistent cough, chest pain, and stomach pain.

## Discussion

This longitudinal cohort study in the Belgian adult population aimed [1] to assess the impact of a SARS-CoV-2 infection and PCC on HRQoL at the time of infection and after three months, and [2] to identify the sociodemographic and clinical factors associated with the evolution of HRQoL following the infection. At the population level, this study found a significant decline in HRQoL during the SARS-CoV-2 infection in comparison to the situation before, but no meaningful clinical difference was detected three months after the infection. At the individual level, we found that PCC (i.e. having at least one symptom related to the SARS-CoV-2 infection three months afterwards) was an important factor associated with a significant decline in HRQoL following the infection. Other studies have shown that PCC leads to poor HRQoL in people who suffer from it [24, 34–37]. A recent meta-analysis on twelve studies on HRQoL among people with PCC (follow-up time from infection ranging from 30 to 180 days) found that 41.5% had problems in the dimension of pain/discomfort, 37.5% in the dimension of anxiety/depression, 36% in the dimension of mobility, 28% in the dimension of usual activities, and 8% in the dimension of self-care [13]. In this study, these percentages were relatively similar and respectively 49%, 40%, 18%, 21%, and 3%. These figures show that a significant proportion of people with PCC have problems in the different dimensions of their quality of life, which poses public health challenges. The negative impact of PCC on HRQoL is explained, among other things, by the number and heterogeneity of its symptoms and their impact on daily life. For example, one study found that some people had to reduce their work hours because of persistent COVID-19 symptoms [38]. This study found that the most frequent self-reported symptoms of PCC were fatigue/exhaustion (21%), headache (11%), memory problems (10%), loss of smell and/or taste (10%), shortness of breath (9%), and sleeping problems (8%). These results are consistent with other studies which found that the most common symptoms of PCC were fatigue, dyspnoea, headache, and attention disorders [8, 27, 39]. In addition, this study showed that the main symptoms of PCC with a significant negative impact on HRQoL were shortness of breath, memory problems, muscle pain, fatigue/exhaustion, and chest pain. The dimension of HRQoL most negatively affected by PCC was pain/discomfort, also negatively associated with the following symptoms: headache, joint pain, dizziness, persistent cough, joint pain, and stomach pain. Although there is still debate about the exact mechanisms behind these PCC symptoms, their negative impact on people’s HRQoL is clear. Other studies have shown that the symptoms of PCC have a negative impact on the quality of life of those who suffer from it [34, 35, 40, 41]. While each symptom independently can have a significant impact on the quality of life, people with PCC often tend to have a combination of symptoms. An important element and challenge in the care of people with PCC is therefore the multidisciplinary approach [42].

Regarding sociodemographic and clinical factors, this study found that women, people with a lower educational level, people with a chronic disease, and people with obesity were more likely to experience a decline in HRQoL following their SARS-CoV-2 infection. These results are consistent with other studies that have shown that women, people with a lower educational level, and people with diabetes or obesity had lower levels of quality of life following SARS-CoV-2 infection [34, 43]. Regarding the greater decline in HRQoL in women, a previous study hypothesized that they may be more aware and attentive to their health in comparison to men, and therefore more concerned about their COVID-19 infection and its impact [34]. Regarding other variables related to COVID-19, this study found that having an increasing number of acute COVID-19 symptoms and being hospitalised due COVID-19 were associated with a decline in HRQoL. These two variables are related to the severity of the infection and other studies have similarly found that COVID-19 severity was negatively associated with HRQoL [35, 44]. Indeed, the severity of the disease has a direct negative impact on physical health and daily life, but also on mental health. For example, some studies have shown that being hospitalised for COVID-19 was a risk factor for mental health problems like post-traumatic stress disorder [10, 45]. Finally, this study found that people without COVID-19 vaccination at the time of infection were more likely to experience a decline in HRQoL compared to people with a complete primary schedule. This result can be explained by the positive impact of vaccination on the severity of acute COVID-19 symptoms and morbidity [46], thus limiting the detrimental impact on HRQoL.

## Limitations

This study has several limitations. The main limitation is the selection bias due to the design of the study. As previously explained, the published study protocol [18] showed that the proportion of people between 46-65 years, of women, and of people reporting at least one acute COVID-19 symptom was higher among cohort participants than in the eligible population, resulting in initial sample selection bias. Therefore, post-stratification weights were used to adjust for the distribution of the eligible population in terms of age, sex, and acute COVID-19 symptoms. However, no information was available on the proportion of PCC in the eligible population and this proportion may be underestimated (e.g. people with PCC may not be in good enough condition to respond to the survey) or overestimated (e.g. people without persistent symptoms may place less emphasis on completing the survey) due to study design. Second, although we used a matched-control group of the 2018 general Belgian population not infected with SARS-Cov-2, this control group was not exposed to the global health crisis and to the measures taken to limit the spread of the virus. Crisis and measures that independently of a COVID-19 infection can have effects on the HRQoL of the general population. In addition, PCC symptoms are common to many other diseases and infections that affect the general population and we do not have information on the frequency of these symptoms in the general population not infected with COVID-19. Although participants self-reported that these symptoms were related to their COVID-19 infection, we cannot perform sensitivity analyses with a control group. Therefore, other studies should be carried out with a control group. Finally, this study followed participants three months after their infection, but further studies should be performed with a longer follow-up period.

## Conclusion

This study highlighted the negative impact of a SARS-CoV-2 infection and PCC on the HRQoL of individuals, respectively in the short (i.e. during the acute phase) and medium term ( i.e. three months later). At the population level, this study found a significant decline in HRQoL during the SARS-CoV-2 infection in comparison to the situation before, but no meaningful decline three months after the infection, except among people reporting PCC. Several symptoms of PCC had a negative impact on the different dimensions of HRQoL such as shortness of breath, memory problems, muscle pain, fatigue/exhaustion, headache, and joint pain. With the high number of people infected with SARS-CoV-2, the impact PCC on HRQoL is an important public health issue. However, our understanding of the aetiology and mechanism of PCC is still in its infancy. Given the heterogeneous symptoms of PCC, further research may assess the effectiveness of a multidisciplinary care approach to limit its negative impact on quality of life.

## Supplementary Information

**Additional file 1.** 

## Data Availability

The data of this study are available from the corresponding author upon reasonable request.
