# Supplementary material for: Post COVID-19 condition and health-related quality of life: a longitudinal cohort study in the Belgian adult population
Source: BMC Public Health. 2023 Jul 27;23:1433. doi: 10.1186/s12889-023-16336-w (PMC10373376; doi:10.1186/s12889-023-16336-w)
Supplement: Supplementary file 1 — Additional file 1. [file 12889_2023_16336_MOESM1_ESM.docx]

**Health-Related Quality of Life and Long COVID Questions from the Baseline and Follow-up Questionnaire**

**BASELINE QUESTIONNAIRE**

Quality of life

**Under each heading, please tick the ONE box that best describes your health BEFORE your COVID-19 infection.**

**Mobility**

Please choose **only one** of the following:

- I had no problems in walking about
- I had slight problems in walking about
- I had moderate problems in walking about
- I had severe problems in walking about
- I was unable to walk about

**Self-care**

Please choose **only one** of the following:

- I had no problems washing or dressing myself
- I had slight problems washing or dressing myself
- I had moderate problems washing or dressing myself
- I had severe problems washing or dressing myself
- I was unable to wash or dress myself

**Usual activities (e.g. work, study, housework, family or leisure activities)**

Please choose **only one** of the following:

- I had no problems doing my usual activities
- I had slight problems doing my usual activities
- I had moderate problems doing my usual activities
- I had severe problems doing my usual activities
- I was unable to do my usual activities

**Pain/discomfort**

Please choose **only one** of the following:

- I had no pain or discomfort
- I had slight pain or discomfort
- I had moderate pain or discomfort
- I had severe pain or discomfort
- I had extreme pain or discomfort

**Anxiety/Depression**

Please choose **only one** of the following:

- I was not anxious or depressed
- I was slightly anxious or depressed
- I was moderately anxious or depressed
- I was severely anxious or depressed
- I was extremely anxious or depressed

**Now, under each heading, please tick the ONE box that best describes your health TODAY .**

**Mobility**

Please choose **only one** of the following:

- I have no problems in walking about
- I have slight problems in walking about
- I have moderate problems in walking about
- I have severe problems in walking about
- I am unable to walk about

**Self-care**

Please choose **only one** of the following:

- I have no problems washing or dressing myself
- I have slight problems washing or dressing myself
- I have moderate problems washing or dressing myself
- I have severe problems washing or dressing myself
- I am unable to wash or dress myself

**Usual activities (e.g. work, study, housework, family or leisure activities)**

Please choose **only one** of the following:

- I have no problems doing my usual activities
- I have slight problems doing my usual activities
- I have moderate problems doing my usual activities
- I have severe problems doing my usual activities
- I am unable to do my usual activities

**Pain/discomfort**

Please choose **only one** of the following:

- I have no pain or discomfort
- I have slight pain or discomfort
- I have moderate pain or discomfort
- I have severe pain or discomfort
- I have extreme pain or discomfort

**Anxiety/Depression**

Please choose **only one** of the following:

- I am not anxious or depressed
- I am slightly anxious or depressed
- I am moderately anxious or depressed
- I am severely anxious or depressed
- I am extremely anxious or depressed

© EuroQol Research Foundation. EQ-5D™ is a trade mark of the EuroQol Research Foundation. UK (English) V 1.0. This is a modified EQ-5D.

**FOLLOW-UP QUESTIONNAIRE**

Quality of life

**Under each heading, please tick the ONE box that best describes your health TODAY .**

**Mobility**

Please choose **only one** of the following:

- I have no problems in walking about
- I have slight problems in walking about
- I have moderate problems in walking about
- I have severe problems in walking about
- I am unable to walk about

**Self-care**

Please choose **only one** of the following:

- I have no problems washing or dressing myself
- I have slight problems washing or dressing myself
- I have moderate problems washing or dressing myself
- I have severe problems washing or dressing myself
- I am unable to wash or dress myself

**Usual activities (e.g. work, study, housework, family or leisure activities)**

Please choose **only one** of the following:

- I have no problems doing my usual activities
- I have slight problems doing my usual activities
- I have moderate problems doing my usual activities
- I have severe problems doing my usual activities
- I am unable to do my usual activities

**Pain/discomfort**

Please choose **only one** of the following:

- I have no pain or discomfort
- I have slight pain or discomfort
- I have moderate pain or discomfort
- I have severe pain or discomfort
- I have extreme pain or discomfort

**Anxiety/Depression**

Please choose **only one** of the following:

- I am not anxious or depressed
- I am slightly anxious or depressed
- I am moderately anxious or depressed
- I am severely anxious or depressed
- I am extremely anxious or depressed

© EuroQol Research Foundation. EQ-5D™ is a trade mark of the EuroQol Research Foundation. UK (English) V 1.0. This is a modified EQ-5D

Long COVID

**Within the last seven days have you had any of these symptoms? (that you did not experience before onset of your COVID-19 illness)**

Please choose **all** that apply:

- No symptoms
- Fatigue/exhaustion
- Headache
- Memory problems
- Loss of smell
- Loss of taste
- Muscle pain
- Shortness of breath
- Sleeping problems
- Joint pain
- Dizziness
- Palpitations
- Persistent cough
- Constipation
- Problems seeing
- Chest pain
- Ringing in ears
- Tingling feeling
- Loss of appetite
- Stomach pain
- Skin rashes
- General malaise
- Weight loss
- Confusion
- Problems speaking
- Problems swallowing
- Swelling/oedema
- Incontinence
- Others
